# Immune cell type, cell activation, and single cell heterogeneity revealed by label-free optical methods

**DOI:** 10.1038/s41598-019-53428-3

**Published:** 2019-11-19

**Authors:** Nicolas Pavillon, Nicholas I. Smith

**Affiliations:** Biophotonics Laboratory, Immunology Frontier Research Center (IFReC), Osaka University, Yamadaoka 3-1, 565-0871 Suita, Osaka Japan

**Keywords:** Imaging and sensing, Predictive markers, Cellular imaging, Cell death and immune response, Monocytes and macrophages

## Abstract

Measurement techniques that allow the global analysis of cellular responses while retaining single-cell sensitivity are increasingly needed in order to understand complex and dynamic biological processes. In this context, compromises between sensitivity, degree of multiplexing, throughput, and invasiveness are often unavoidable. We present here a noninvasive optical approach that can retrieve quantitative biomarkers of both morphological and molecular phenotypes of individual cells, based on a combination of quantitative phase imaging and Raman spectroscopy measurements. We then develop generalized statistical tools to assess the influence of both controlled (cell sub-populations, immune stimulation) and uncontrolled (culturing conditions, animal variations, etc.) experimental parameters on the label-free biomarkers. These indicators can detect different macrophage cell sub-populations originating from different progenitors as well as their activation state, and how these changes are related to specific differences in morphology and molecular content. The molecular indicators also display further sensitivity that allow identification of other experimental conditions, such as differences between cells originating from different animals, allowing the detection of outlier behaviour from given cell sub-populations.

## Introduction

Cells have numerous traits that can be used to identify them: genetic, epigenetic, behavioural, compositional, or morphological features can all be used to define the phenotype of a given cell. As the discrimination capabilities of measurement systems improve, newly defined cell sub-types can emerge^1,2^. Predictive biomarkers are also increasingly important for customized therapy^3^. However, with increased sensitivity in the detection of specific markers, the inherent cell heterogeneity becomes more important^4^, and methods to assess the meaning and significance of such variability become increasingly needed^5^. The implication of such cellular heterogeneity is particularly important in the field of immunology, where diversity is at the core of an efficient immune defence system^6^. Several methods have been reported in recent years that can reveal different response patterns among individual cells^7^. In particular, fluorescence has emerged as a tool of choice in the field of proteomics, enabling the study of single-cell level intracellular protein expression^8^, or allowing the study of multiple effector molecules for deeper assessment of cellular heterogeneity through massively multiplexed systems^9,10^.

However, fluorescence-based methods modify the cell, and are generally limited to a relatively low number of targets, albeit with high specificity. Furthermore, most markers rely on surface proteins, since measuring intracellular molecules and dynamics requires more invasive procedures such as transfection^11^. On the other hand, different emerging techniques such as single-cell sequencing can provide highly specific information, but at the cost of being destructive^12^. A single-cell measurement system that can simultaneously examine a wide range of cell molecules, while remaining non-invasive should therefore have significant advantages in studying small cellular population phenotypes.

As a step towards this goal, we recently demonstrated the use of all-optical multimodal label-free analysis to characterize individual cell activation states^13^. This system is based on quantitative phase imaging (QPI)^14^ and autofluorescence, that provide images from which label-free morphological indicators can be extracted, and is combined with Raman scattering spectroscopy^15^. Both techniques have recently been used with multivariate analysis or machine learning approaches to develop label-free biomarkers, either through morphological^16–20^ or molecular^21–23^ indicators.

Having previously demonstrated the ability to discriminate between activation states of a macrophage cell line (Raw264), in this report we show that primary cultured peritoneal cavity macrophages—either resident or elicited populations^24^—exposed to lipopolysaccharide (LPS) manifest phenotypic changes in both morphology and molecular content that can be discerned by our label-free optical system. Such experiments involve several sources of variability, either controlled, such as the LPS exposure and the choice of extraction method, or uncontrolled, such as culturing conditions, drug response level, etc. The single-cell heterogeneity within any of these cell populations can then be compared to any other population by multivariate analysis, where high-level indicators such as the ones derived from label-free measurements will be influenced by all these factors at different degrees. Simple principal component analysis (PCA) shows that the largest contributions to the variance in the label-free data comes from the activation state and the cell type, which are both highly relevant phenotypic parameters. Other more subtle morphological or molecular features also emerge as characteristics of cell populations, and in some cases highlight a possible split, leading to sub-populations of potential interest for further study.

In order to systematically study more subtle heterogeneities, we create a generalized method for assessing the effects of both controlled and uncontrolled variations within cell populations over multiple days, animals and culture dishes by using the F-test values, common in analysis of variance (ANOVA). It is possible through this approach to identify cells from individual mice as having distinct populations, and some mice could be identified as the source of outlier populations, although the overall variance from these experimental parameters is small compared to the main phenotypic features originating from cell type or activation.

Machine learning is not required to perform such exploratory analyses, but when supervised learning is applied in addition, high performances in cell classification can be achieved, and predictive label-free indicators can be derived to assess both the cell type as well as LPS-induced activation. The feature vectors generated with our system are able to also detect whether the cell is from the population of resident or recruited peritoneal cavity macrophages, which have recently been shown to be separate sub-populations with different progenitors^25^ and function^26^.

## Results

### Data organization

To assess the main factors influencing cellular variability, we selected different types of macrophage cells that we measured in control and stimulated environments, providing different controlled conditions that we employ to study their influence on cellular variability. In particular, we use the macrophage-like cell line Raw264, as well as primary macrophage cells extracted from the peritoneal cavity, which are macrophages fully differentiated *in vivo*, and known for having a large inner population variability^26^. We also study both resident peritoneal macrophages (RPM), which are composed of the inherent population present in the peritoneum, and elicited macrophages (EPM) that are recruited into the peritoneal cavity upon external stimulation. Elicited macrophages have been employed in numerous studies to circumvent the poor yield of RPM and ensure a sufficient amount of cells to study while minimising animal usage^24^, although it has also been demonstrated recently that the extracted cells can be functionally different^25,26^. Furthermore, cells are also stimulated with lipopolysaccharide (LPS), a compound that is found at the surface of Gram-negative bacteria.

**Figure 1:**
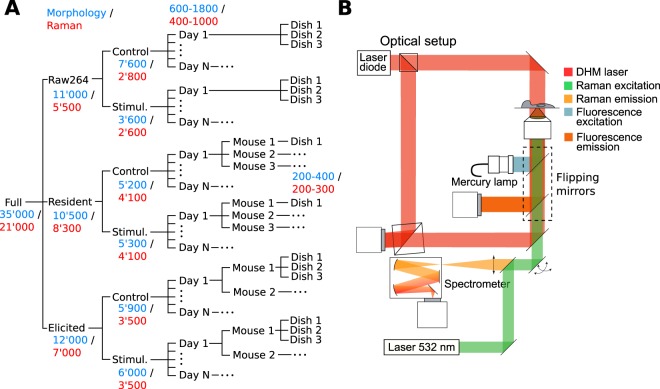
(**A**) Data structure showing the different conditions and sample sizes at each level of the experiment for both morphological and Raman parameters, respectively. (**B**) Measurement setup providing label-free quantitative phase imaging as well as single-cell Raman spectra.

These experimental settings, composed of 3 cell types and 2 conditions (Control and LPS) yield 6 main sets of controlled conditions as shown in Fig. 1A, where measurements have been approximately evenly distributed between them. To understand how uncontrollable variability might affect the results, the measurements are performed over several days, with cells plated onto different dishes to account for differences in cell culture, where 200–400 cells are measured per dish. This ensures that each data subset can be considered as statistically relevant, with 3–6 dishes per condition and day. In case of RPM, the low yield per mouse implies that cells extracted from one mouse could be employed to prepare only one set of experiments, while the larger amount of cells in case of EPM allowed the preparation of several dishes.

### Label-free measurements

The measurements are performed through a label-free multimodal optical platform previously reported^13,27^, described in more details in the Methods, and illustrated in Fig. S1. Briefly, we combine the imaging information from quantitative phase imaging (QPI) recorded with off-axis holography and auto-fluorescence (AF), along with Raman spectroscopy, to retrieve single-cell level indicators enabling the analysis of cellular responses and conditions, as shown in Fig. 1B. QPI and Raman spectra are acquired simultaneously with an approach based on spectral separation^27^. To increase measurement throughput, Raman spectra from the cells present in the QPI field of view are recorded as point spectra, where the excitation laser is rapidly scanned within each cell body to retrieve an averaged spectrum more representative of the whole cellular content^28^. AF images are recorded sequentially in a standard epi-fluorescence system provided by a set of flipping mirrors (see Fig. 1B).

The imaging data is then reduced by registering both QPI and AF images together, and segmenting individual cells to extract various parameters based on the cellular morphology through the use of different modules of the *CellProfiler* program^29^. As it has been observed that data from morphology and spectra provide complementary information^13^, they are here analysed separately.

**Figure 2:**
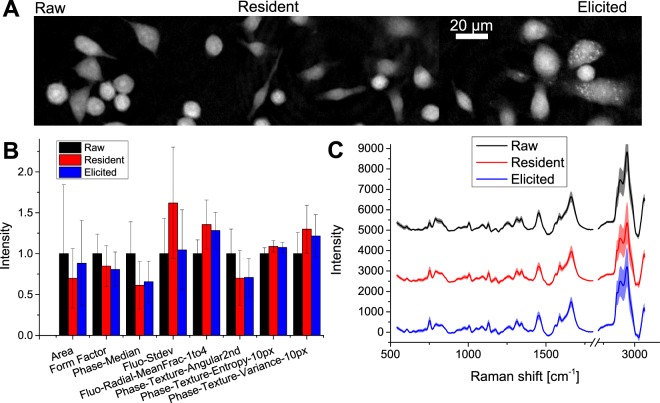
(**A**) Typical QPI images for all cell types. (**B**) Selected average morphological parameters extracted from *CellProfiler* (see full list in Table S3) that display the most significant differences between cell types, where values have been normalized based on the ones of Raw264 (errors bars indicate standard deviation). (**C**) Average cellular Raman spectrum, per cell type. Standard deviation is represented by the shaded colour regions.

Typical data is shown in Fig. 2, where average signals from the whole dataset (N = 20,798) for each cell type are displayed. While Raman spectra are very similar between cell types (see Fig. 2C), there are small differences that can be visually identified, such as stronger peaks at 750, 1050 and 2854 cm^−1^ for peritoneal macrophages compared to Raw, but a weaker main CH peak at 2935 cm^−1^. In addition to differences in the values themselves, it is also possible to identify differences in the variance, where for instance peritoneal macrophages appear to have larger standard deviations in the CH stretching region than Raw cells. Such comparisons can also be performed on the cell morphological phenotypes, where the overall morphology appears to be quite different between cell types for some cell sub-populations, as shown in Fig. 2A for representative QPI images. This is also shown when observing some average values extracted from segmented cells, which are a small subset of the 301 morphological values (see Fig. 2B, and the Methods section for the derivation procedure). It is possible, for instance, to see that peritoneal cells are more elongated (smaller Form factor) and induce a smaller median phase shift than Raw cells, which is consistent with the representative QPI images, or that RPM have a much larger variation in the auto-fluorescence signal compared to both EPM and Raw cells.

### Phenotype and molecular content highlight different features

**Figure 3:**
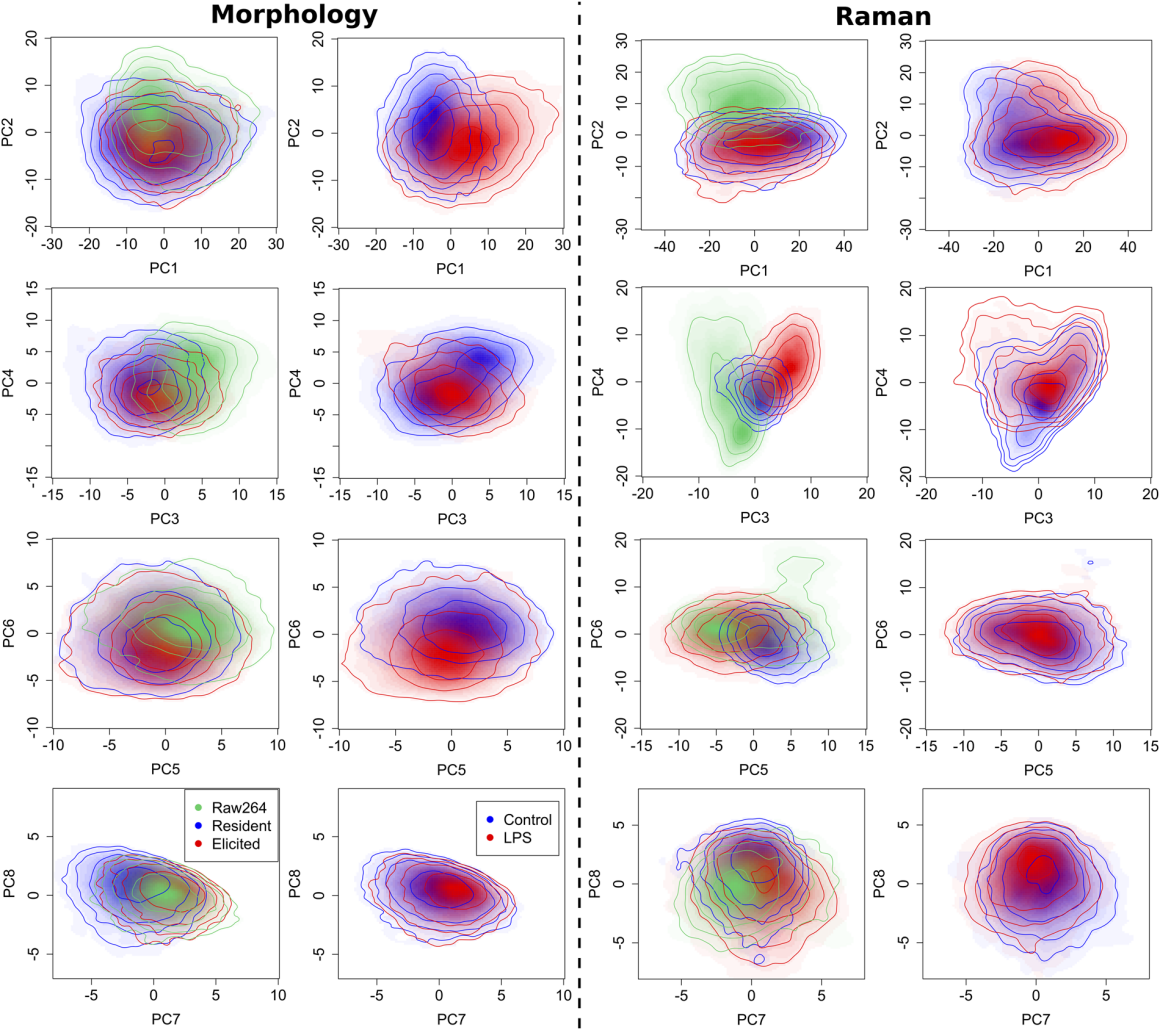
Principal component analysis score plots, where the colours and corresponding contour lines represent the data density, for PC 1–8 of morphology and Raman data, respectively, showing the contribution of each cell type and drug response to data variability.

To further analyse the data, we then apply PCA to derive the main influences on data variability. By considering the contribution to variance from each principal component (see Fig. S2), it is possible to identify a shoulder at PC8 and PC7 for morphology and Raman, respectively, where the contribution falls below 2%. We therefore restrict our analysis to the first 8 principal components. The score plots (represented as density maps) are shown in Fig. 3, where the same plots are displayed twice, first by colouring the distribution of the three cell types observed in the experiments, and a second time by showing the distributions of both control and stimulated cells.

It is possible to see that these two main sets of conditions (cell type and stimulation) are both significantly separated by both sets of variables, but by different components. One first striking point is the similarity of the overall distribution of the score plot for the first two PCs for both morphology and Raman, where PC1 essentially differentiates control from stimulated cells, while PC2 separates Raw and peritoneal macrophages. Even though morphology and Raman are independent and measure different aspects of cell phenotype, the strongest variance in both types of measurement is remarkably similar and separates cells based on their level of stimulation (PC1) or cell type (PC2). When combined, PC1 and PC2 contribute for 43.43% and 50.48% of the variance in morphology and Raman, respectively. This implies that approximately half of the variance is related to the separation of the main experimental factors under consideration here (cell type and cellular state).

The score plots are then rather different for higher PCs, where differences between the two label-free modes appear more clearly: morphology mostly differentiates control vs. LPS, while Raman is more specific on cell types, as summarised in Table 1. In particular, Raw is clearly distinct from peritoneal cells in both data sets as it is singled out by several PCs, while the difference between RPM and EPM is picked only in higher PCs; PC7 in morphology partly separates RPM, while PC3,4 in Raman clearly separate all cell types.

**Table 1:** Main features highlighted in the score plots of each principal component (see Fig. 3), along with the interpretation for each corresponding loading vectors (see Figs S3 and S4), separated in the case of Raman between positive (+) and negative (−) features.

	Main separation	Interpretation
	Morphology	
PC1	Control vs. LPS	Strong shape and texture contributions
		Strong QPI/AF correlation (0.96)
PC2	Raw vs. Primary	Weak shape, overall QPI/AF contributions
		Strong QPI/AF correlation (0.95)
PC3	Raw vs. Primary	Strong QPI/AF texture, QPI intensity
		QPI/AF anti-correlation (−0.53)
PC4	Control vs. LPS	Overall parameters
		Positive QPI/AF intensity
PC5	Raw vs. Primary (weak)	Weak shape, strong texture
		QPI/AF anti-correlation (−0.55)
PC6	Control vs. LPS	Strong shape, QPI/AF radial
		Strong QPI/AF correlation (0.84)
PC7	Resident vs. else	Weak shape
		Negative AF intensity
PC8	Unclear	Strong QPI radial
	**Raman**	
PC1	Control vs. LPS	(+) General cell content
PC2	Raw vs. Primary	(+) Proteins
		(−) Cytochrome C
PC3	Cell types	(+) Cytochrome C
		(+) Unsaturated lipids
PC4	Cell types and stimulation	(+) Lipids
		(−) Proteins
PC5	Resident vs. else	(+) Cytochrome C
		(+/−) DNA/RNA residues
PC6	Raw sub-population vs. else	(+) CH stretching, amino-acids
PC7	Cell types (weak)	(+) Proteins
PC8	LPS (weak)	(+) CH stretching

It should also be noted at this point that the analysis performed here is purely based on intrinsic variance of the data through PCA, without supervised learning. The classification power of the studied parameters might then be different from these observations, if supervised learning methods were employed.

The loading vectors corresponding to each principal component are shown in Figs S3 and S4, for morphology and spectra, respectively. The morphological loading vectors are all composed of a mixture of parameters, making the interpretation difficult, although some main tendencies (mostly shape, overall intensity, etc.) can be extracted, and are summarised in Table 1. Interestingly, the vector with the clearest features, PC8, which are concentrated on radial phase features, is also the one providing the least clear separation between experimental conditions. This shows that the ability of separating between cell classes requires a mixture of morphological variables. Another striking point is that there are strong correlations between QPI and AF parameters, where most vectors are either highly correlated (PC1,2,6) or anti-correlated (PC3,5), while the others display strong directionality (overall positive intensity parameters in PC4, for example).

The Raman loading vectors are displayed with multiplication by the PCA scaling factors so that they are similar to actual Raman spectra, with their main contributions summarised in Table 1. In details, PC1 is very similar to typical spectra recorded through hybrid scanning^28^, and contains features from general cellular components. PC2 is more specific, with negative peaks that can be attributed to cytochrome C (750, 1129, 1586 cm^−1^)^30^, while positive bands are essentially representative of protein secondary structure (Amide I, 1650, 1684 cm^−1^ and Amide III 1245, 1328 cm^−1^)^31^. PC3 contains similar peaks of cytochrome C, but appears combined with lipids bands (CH_2_, 2854 cm^−1^ and CH_3_, 2890 cm^−1^)^32^, along with unsaturated lipids (C = C, 1661, 3014 cm^−1^)^33^.

The following vectors become more complicated, where PC4 is mostly composed of lipids (1438, 2850, 2890 cm^−1^), but with differential features in the CH stretching region (2949, 2979 cm^−1^), and negative CH stretching (1340 cm^−1^), Amide I (1676 cm^−1^) and phenyl ring (1009 cm^−1^), indicative of proteins. PC5 contains again cytochrome C features, along with ring stretching features (783, 1150–1370 cm^−1^) indicative of nucleic acids^34,35^, negative lipids (strong 2854/2890 cm^−1^ ratio), and opposite unsaturated fatty acids (2923 cm^−1^). PC6 contains general CH stretching, with some positive features in amino-acids residues regions (744–876 cm^−1^)^31^, and especially tryptophan (756, 1009, 1557 cm^−1^)^36^. PC7 is mostly indicative of proteins with CH stretching bands (1440, 2848, 2918 cm^−1^)^37^, and Amide I (1650 cm^−1^) and Amide III (1304 cm^−1^). PC8 is mostly composed of high wavenumber contributions.

### Different cell types have different variability levels

Interestingly, it is also possible to identify that Raw, a population composed of clone cells, appears to be overall less heterogeneous than peritoneal cells both in morphology and cellular content, as illustrated in PC1,2, where the distribution of Raw is smaller than RPM and EPM, except for PC2 of Raman, where Raw seems slightly wider. On the other hand, there seems to be a sub-population of Raw in higher PCs of Raman (PC4–6).

**Figure 4:**
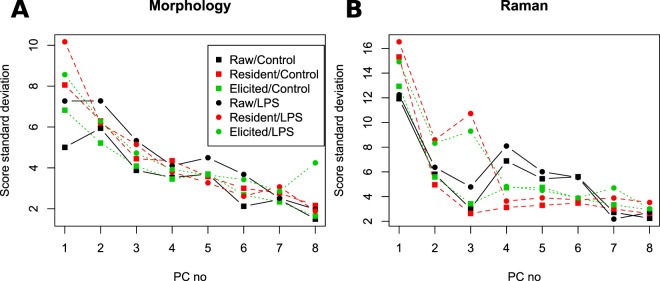
Standard deviation of scores for each PC, divided by cell type and stimulation condition.

In order to quantify the variability of the different observed species, we compute the standard deviation of the PCA scores for each PC (see Fig. 4). It can be identified that Raw indeed has less variation in most morphology-related PCs (see Fig. 4A), where it becomes especially apparent because the analysis is performed here separately on control and stimulated cells. Furthermore, RPM cells appear to have more heterogeneous morphologies than EPM cells. On the other hand, the variability in spectral features seems more comparable, but with RPM being more variable than the two other cell types. A sub-population is also present in PC4–6 (see Fig. 3), which increases the variability of Raw. The exposure to LPS also consistently increases morphology variability for all cell types, and stimulated peritoneal macrophages appear to have significantly more variable spectral features in PC2,3, and to a lower extent PC1. It should be however noted that while the study through standard deviation is useful to extract main tendencies, it can lead to misinterpretations, as it assumes that the distributions can be approximated as Gaussian, which is clearly not the case for some of the score plots (see Fig. 3).

### Label-free parameters provide reliable classifiers of activation and cell type

The multivariate analysis as performed above through PCA can provide important insight into the data structure, but it does not create sufficient separation between species to enable classification. It is however also possible to develop models based on supervised learning in order to account for the inherent variability in the data and actively distinguish the selected experimental conditions. We employ here models based on penalised logistic regression applied on either morphological or Raman data (see details in Methods), which have been demonstrated to be able to detect the LPS-induced activation state of Raw cells^13^. The logistic distribution is ideal to distinguish between binomial classes, and the model employs an L1 penalty term that shrinks the coefficients of variables that do not independently contribute to the separation power of the classification model^38^. This approach effectively reduces the amount of variables used in the model, which contributes to its stability. We employed multinomial models with Raman parameters to distinguish between cell types and activation states, yielding 6 separate categories, where each specific category is separated from the other 5 classes by a dedicated model.

The models are trained with 15,000 random samples from all measurements, corresponding to approximately 50% and 75% of the data for morphological and Raman variables, respectively. The models are then employed to predict the condition of the remaining data, as shown in Table 2.

**Table 2:** Confusion matrices for classification predictions. Raman: full multinomial separation between the 6 species. Morphology: separate models to classify stimulation condition and cell type, respectively.

Raman		Raw		Resident		Elicited	
N = 5,798		Control	LPS	Control	LPS	Control	LPS
**Raw**	Control	96.74	3.32	0.086	0.176	0	0
	LPS	3.02	96.26	0	0.088	0	0.101
**Resident**	Control	0	0.138	90.83	7.92	1.47	0.201
	LPS	0	0	4.88	93.84	0.21	1.11
**Elicited**	Control	0	0	1.63	0.176	88.04	9.38
	LPS	0.121	0.138	0	1.23	11.75	87.1
**Morphology**					**Raw**	**Resident**	**Elicited**
**N** = **18,652**		**Control**	**LPS**	**Raw**	92.3	0.928	6.36
	**Control**	88.5	14.5	**Resident**	0.922	86.5	11
	**LPS**	11.7	85.3	**Elicited**	7.41	15.3	79.7

Overall, the models can achieve sensitivities in the range of 87–97%, with comparable specificities, showing that is possible to create reliable models, even when including data across multiple conditions and including uncontrollable or unknown sources of variation (such as experiments being performed on different days, dishes, etc.). Interestingly, the errors are strongly correlated, where most of the misclassifications correctly identify the cell type but misidentify the activation state, or correctly finds the activation but confuses peritoneal cells. It is also possible to see that the identification of the Raw cells is especially accurate (~96–97%), which is consistent with the previous analysis not using supervised learning, where the Raw cell line was identified as being more different than the other cell types in the PCA score plots (see Fig. 3). Interestingly, with the addition of supervised learning, the Raman indicators are able to distinguish activation accurately, despite the separation not being obviously visible when simply considering the PCs displayed above. This shows that the higher order PCs from Raman measurements, although only responsible for a small amount of the total variation in the data, can also have significant discrimination powers and may serve as useful biomarkers or act as a basis for further study into the differences between cell types.

In the case of morphological parameters, it was not possible to create stable multinomial models able to accurately predict the 6 different classes. However, it is possible to create models that can predict either the activation state or cell type, with 85–88% and 80–92% sensitivity, respectively, based on morphology. While the overall sensitivity is lower than in the Raman case, the overall behaviour is similar, with Raw cells being more different than peritoneal macrophages, and with the cell type being easier to differentiate than the activation state. It also shows that while the morphological phenotype can distinguish between resting and activated cells, or between Raw, RPM and EPM, it cannot alone disentangle the two effects.

It should also be noted that the label-free parameters (Raman and morphology) have been kept separate throughout this article to simplify the data interpretation and the study of the various parameters. It is however also possible to combine the two data sets by pairing the measurements. In that case, the size of the data set is slightly reduced since only cells that were measured in both morphological and Raman modes are used. This still leaves a significant (N = 17,507) amount of cells, out of which 10,000 (57%) are used for training the paired data model. A significant improvement in the classification can then be obtained, with sensitivities in the range of 91.5–99% (see Table S1) for multi-class models. This implies that while the morphological parameters are not specific enough to enable full classification by themselves between the 6 species, they can provide additional information to the spectral measurements to significantly refine the statistical models.

### Quantitative assessment of the influence of the experimental factors

One important question is the degree to which different sources of variation in the data (such as known/controlled or unknown/uncontrolled experimental factors) can affect outcomes, and whether the single-cell heterogeneity is biologically significant^5^. Overall, the previous section showed that in this experiment, the main variation across the whole dataset is due to the parameters under investigation (i.e. cell type and/or cell activation). This implies that the study is robust in terms of uncontrolled parameters. However, it is still of interest to determine to what degree other factors can be seen to affect the results by performing further analysis on the dataset. As shown in the previous sections, it is complicated to analyse the influence of the different factors in highly multivariate settings. Even in the case of strong data reduction, as performed by PCA, only the cell type and activation state effects emerged, as clear separating features, while there are also other potential influences such as variations between the individual dishes used for measurements, or individual animals employed for cell extraction, as shown in Fig. 1.

Therefore, in order to assess the potential influence of these multiple experimental factors, we employ a metric that can be used to quantify the respective separating power of all PCs on the different experimental factors, thus allowing a semi-automated analysis of the numerous combinations. We employ here the F-test statistic, which is defined as the ratio of the *between-group* variance to the *within-group* one^38^. We then use the value of the F-test as a quantitative indicator of how well a given PC can separate groups based on a given experimental factor, allowing us to systematically assess the influence of all components.

**Figure 5:**
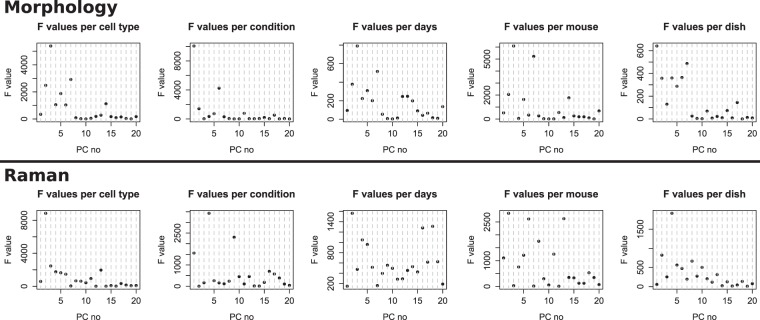
F-test values for each known experimental parameter in the data structure (cell type, stimulation, days, animals and dishes) for both morphology and Raman variables.

This analysis, displayed in Fig. 5, confirms the main tendencies observed in Fig. 3. It shows that morphology mainly differentiates control and stimulated cells on PC1,6, and also distinguishes to a lesser extent between cell types in PC2,3,7. On the other hand, spectral indicators are more efficient at separating cell type in particular through PC2,3, but can also separate the stimulation with PC4,9.

The quantitative values in Fig. 5 can also be used to study the influence of other parameters which might have undesired influence on the data variability. In the case of morphology, it is possible to see that the F-test values computed between days, mice or dishes are either of much lower intensity than the main parameters of cell type or culturing condition, and/or follow a pattern that is very close to the one of cell types. This shows that the differences which appear to be originating from these factors are due to the fact that only one cell type is measured per single day or mouse.

To further confirm the influence of the different parameters, it is also possible to compute the F-values separately for each cell type. In the case of morphology, this decomposition indeed leads to F-test values that do not exceed 150 for low-order PCs, or values that can be attributed to cell type (see Fig. S5), showing that morphological parameters are only minimally influenced by day-to-day or dish-to-dish variations. This is illustrated in Fig. S6, where scores per dish are shown for representative values of PC1,3, previously attributed to cell type and condition. It can be seen that while there are some differences for particular dishes, the values are overall consistent even within the two PCs having the largest F-test values.

### Spectral parameters are partly influenced by days and dishes

On the other hand, Raman F-test values are larger overall for the days, animals and dishes (see Fig. 5), and the values per PC do not follow a pattern identical to cell types or condition, implying that these undesired parameters have some direct influence on the data variance. In particular, when considering the effect of different days, the first largest F-test values corresponds to PCs that can also be attributed to other effects (PC2, cell type and PC4, condition). However, other PCs (PC5,9, etc.) also have F-test values that are significant. When considering the F-test decomposition by cell type (see Fig. S5), large values are also obtained when comparing days, but they are found only in Raw cells (PC4-5), while peritoneal macrophages have either much smaller values, or larger ones only in higher-order PCs (PC10, and most PCs above 13th).

These variations are shown in Fig. S7 with PC5,10 as a compromise between selecting high F-test values and favouring low-order PCs, so that they represent large data variance. It can be seen that the spectral scores are quite stable day-to-day, as most of the variation induced by days is driven by Raw cells. Two main groups of Raw can be identified, which can be explained by the very large time difference between these two batches of experiments (see Table S2), which are separated by 9 months, time during which consequent readjustments to the experimental system were performed. Significant differences can also be observed for EPM, where some experiments are separated by 3–4 months. These differences are however mostly observed on PC10, which represents only a fraction of the data variance.

Similarly, when considering the influence of dishes on the Raman signals, it is possible to see that most low-order values are quite low (see Fig. S5). However, PC4,6 have relatively larger values for both Raw and RPM. These scores are shown for representative values in Fig. S8, where small dish-to-dish variations can be identified, although these are fairly limited, and even smaller than day-to-day ones.

### Raman features are sensitive to animals

By comparing the F-test values in Fig. 5 for different experimental parameters, the variation from one mouse to another appears larger than variations due to days or dishes. As previously, some PCs can be attributed to other factors such as cell type (PC2), although several PCs have relatively large values (PC6,8,13, etc.). The F-test values separated by cell types (see Fig. S5) also show quite different profiles and larger values for RPM (PC14,17) and EPM (PC8,15).

**Figure 6:**
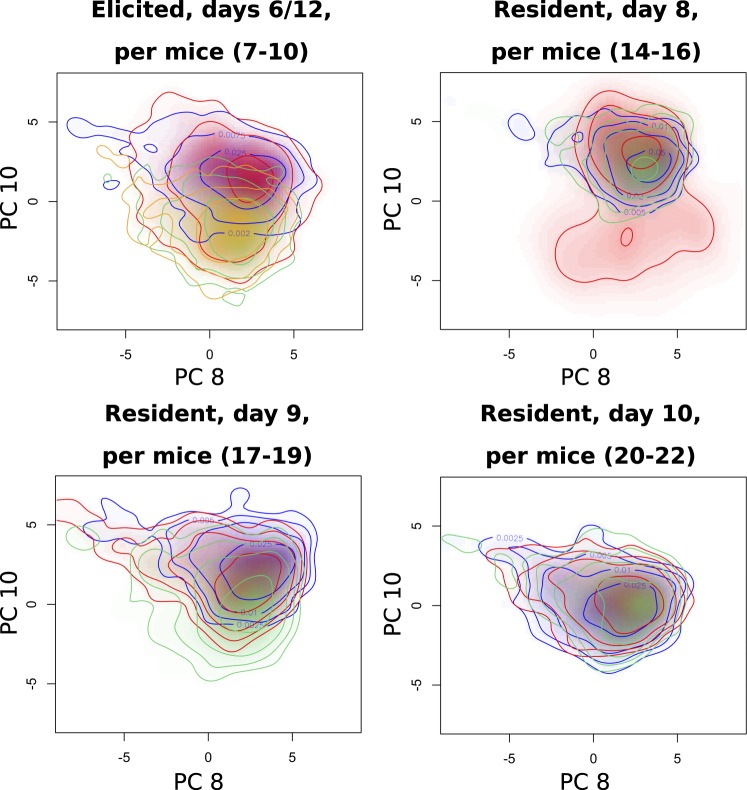
Representative density maps of score plots from Raman parameters where the influence of individual mice can be identified, plotted by cell type and day.

To illustrate this influence, we select the PC having the largest F-test value for each cell type that is within the ten first ones, giving PC 8 and 10 for RPM and EPM, respectively. Representative score plots for these PCs are shown in Fig. 6, where significant differences between mice can be identified. First, it can be seen that overall, RPM appear to have mostly less variation than EPM. There is also some influence from the day of experiment, that affects the average score values such as mice 7–8 and 9–10 for EPM, or mice 17–19 and 20–22 for RPM, although scores are overall fairly stable throughout all days.

When comparing mice, some of the most important differences are not necessarily in the average scores of the cells, but in the fact that certain mouse-derived populations have much larger variations, such as mouse 15 on day 8, or mouse 19 on day 9. This effect is also restricted to RPM where certain populations have these outlier variances. EPM have overall larger variations, but do not display such outliers. All these trends are visible in the representative set of Fig. 6, and are also confirmed when considering the experiments from all the different days (see Fig. S9). Furthermore, similar behaviours could be identified when studying the scores of other PCs having significant F-test values. Taken together, these results show that using label-free analysis of mouse-derived populations makes it possible to identify individual mice that produce cell populations with a variance that could be considered as an outlier.

## Discussion

The different label-free modalities employed in this study to characterise live cells rely on different contrasts, and provide quite complementary information, as illustrated during the analysis of the data, where it was for instance identified that morphological indicators are more sensitive to the LPS stimulation, while spectral ones are primarily influenced by the cell type. These differences are consistent with the fact that all the measurements, namely quantitative phase and auto-fluorescence imaging, as well as Raman spectroscopy, are independent.

However, one striking point is the strong relation between the various measurements, for which very similar trends were identified. The first loading vectors from PCA of morphological parameters showed strong correlation or anti-correlation between QPI and AF. Furthermore, similar trends can be identified from LPS stimulation and cell type in the first score plots of morphology and Raman variables, which account for 40–50% of the data variance. This suggests that these measurements, which rely on different sources of contrast—molecular for Raman, morphological for imaging—are similarly influenced by these external factors. The two measurement indicators are nevertheless very complementary, as further PCA analysis reveals very different information between the available variables. In higher components, morphological indicators mostly highlight differences between stimulated and control cells, while molecular indicators emphasise differences between cell types.

The proposed approach makes it possible to distinguish between all cell types. Furthermore, it appears that Raw cells are strongly separated from peritoneal macrophages both in terms of morphological and molecular phenotype. This can be expected as it is a cell line, which should be less similar than two primary cell types originating from mice of the same species. Smaller differences, although still significant, can also be identified between both types of peritoneal macrophages, which is expected as it has been shown that RPM and EPM are distinct sub-populations that originate from different progenitors^39^.

It also appears that the inner variability within Raw cells is smaller than within peritoneal macrophages, both in terms of phenotype and molecular content. This is consistent with the fact that peritoneal macrophages are known to be quite heterogeneous^40^, while Raw cells have been reported to be functionally stable within a reasonable amount of passage numbers^41,42^. However, the difference in variation is not especially large, showing that even cell lines can have a large inner heterogeneity. This is confirmed by the influence of LPS, which itself increases the inner variability by a comparable amount. It should be noted that inner variability is observed here through global indicators that are influenced by the ensemble of molecules whose influence is studied through multivariate analysis, compared to other studies where the behaviour of very specific molecules was reported^8,9^.

Such a label-free approach can therefore be applied to distinguish between close cell types, and between fine cellular states within these cells. As shown by the loading vectors resulting from PCA, the changes that differentiate between the species are distributed over many different aspects of the phenotype, both morphological and molecular. For instance, texture parameters or protein bands contribute to both the differentiation of cell types or stimulation, showing that an accurate separation involves the combination of multiple variables. This shows that exploratory analysis such as PCA can provide valuable insight about the main influencing experimental factors and about the intrinsic variability in the data. However, if reliable training data is available, other methods based on supervised learning can be developed to also generate stable predictors.

Models created with these label-free indicators can reliably predict either the activation state, or the type at single-cell level. This is particularly true for Raman indicators, which can distinguish 6 cases, composed of 3 cell types and 2 stimulation conditions. Furthermore, pairing morphological and molecular indicators for individual cells identification yields a significant improvement in prediction performances, indicating that they both contain uncorrelated information that can be employed for classification. Interestingly, the high sensitivity (>90%) implies that the vectors are robust to uncontrollable factors such as different days of measurements, or extractions from different animals. The predictors are also robust to impurities, as it is known that cells extracted from the peritoneum are not exclusively macrophages^26^.

It is also interesting to note that such a label-free multivariate approach can provide high classification sensitivities, as other more conventional approaches, such as fluorescence, often require several markers to non-ambiguously identify a specific cell type. For instance, differentiating between RPM and EPM can be typically performed through surface markers such as CD11b, F4/80 and MHC-II^26^. Furthermore, identifying multiple cell types can require additional markers. Raw264 is for example known to express also both CD11b and F4/80 to a significant degree^41^. Similarly, the detection of the activation state can be performed through fluorescence, but requires the staining of intracellular molecules such as NF-*κ*B or iNOS^8^, and is hence destructive.

The multitude of experimental factors, both intended (cell types, stimulation), and unintended (dish or mice variations), cannot easily be analysed even with the data reduction provided by PCA. We therefore used the F-test values as a quantitative indicator of the separating power of each PC for a given parameter, to systematically analyse the influence of each component. This approach makes it possible to disentangle the effect of the different parameters, showing that some of them have effectively no significant influence, or to extract the PCs that have the strongest influence on a given parameter.

The results show that morphological parameters are not significantly influenced by additional factors such as days, dishes or animals. On the other hand, spectral variables display significant changes on higher components depending on days, which increase in case of experiments that are further apart, showing that this depends mostly on instrument drifts, despite the calibrations that are applied to the data. Such an approach might enable the extraction of the features induced by the drift, to further compensate for them. As discussed above, such drifts can also be compensated within supervised learning models.

Raman indicators are also influenced by individual mice, where cells extracted from separate animals display different features. The average scores are fairly consistent in the case of RPM, with cells from specific mice displaying an outlier behaviour. On the other hand, the average EPM scores are more varied, but no outliers can be identified. This might be explained by the fact that EPM result from mice treated with thioglycollate, so that the average changes could indicate a different response to the treatment. On the other hand, RPM are extracted from control mice, so that there is less variation for most cell sub-populations, but some animals with outlier scores may have unknown pre-existing conditions, or may have responded to unintended external conditions.

## Materials and Methods

### Primary cells extraction

C57BL/6N mice were purchased from Japan SLC, and were maintained under specific pathogen-free conditions. All animal experiments were conducted with the approval of the Animal Research Committee of the Research Institute for Microbial Diseases in Osaka University, Japan (approval no H29-02-0), and were conducted in accordance with the guidelines of the Animal Care and Use Committee of Osaka University. Female mice aged between 6 and 9.5 weeks were used for all experiments.

The extraction of peritoneal macrophages is performed through standard procedures^24^. Briefly, to elicit macrophages, 1 mL of a 3% w/v Brewer thioglycollate medium (Sigma-Aldrich) preparation in sterile DI water, aged for at least one month at RT, is injected in the peritoneal cavity, and left to react for 3 days. Mice (with or without injection) are sacrificed, and 10 mL of cold phosphate buffer saline (PBS, Nacalai) with 1% bovine serum albumin (BSA, Sigma-Aldrich) is rapidly injected 2–3 times in the peritoneal cavity, and recovered to be kept on ice. Cells are then centrifuged at 300 ×*g* for 5 minutes, and resuspended in cold culture medium (Dulbecco’s modified Eagle medium (DMEM, Nacalai) supplemented with 10% fetal bovine serum (Gibco) and penicillin/streptomycin (Sigma-Aldrich) with 10,000 units and 10 mg/mL, respectively, diluted at 10 mL/L. Cells are plated at 1–1.5 · 10^5^ cells/cm^2^ on 3.5 cm quartz dishes (FPI) and incubated at 37 °C in a humidified atmosphere containing 5% CO_2_.

Quartz substrates are previously coated with poly-L-lysine (PLL) by immersing the surface in a 0.01% PLL solution (Sigma-Aldrich) during 30 minutes at room temperature (RT). The surface is then washed with deionized (DI) water and left to dry for 2–3 hours at RT before plating the cells.

### Cell culture

Raw264 cells (Riken BioResource Center) are cultured in 10 cm culture dishes and immersed in culture medium, and incubated as stated above. Cells are trypsinysed with a solution containing 0.25% trypsin and 1 mM ethylenediaminetetraacetic acid (EDTA, Nacalai) for approximately 5 min at 37 °C to detach them from the dish. They are then plated on quartz dishes at a density of 0.5 · 10^5^ cells/cm^2^ and then incubated again as described above. Cells from passage number 12 to 18 were used for all experiments.

Then, 6–8 hours after plating, cell cultures (either primary cells or Raw264) are first rinsed with DMEM, which removes non-adherent cells in case of peritoneal cells, and cultures are then immersed in fresh medium containing LPS from E. Coli O111:B4 (Sigma-Aldrich) at 100 ng/mL.

### Cell measurements

After 24 hours of stimulation, cells are washed 2–3 times with PBS supplemented with glucose (5 mM) and MgCl_2_ (2 mM) before measurement.

Cells are measured with a multimodal microscope previously described^13,28,43^, illustrated in Fig. 1. The overall reconstruction and data processing procedure is also summarized in Fig. S1. Briefly, samples are imaged with a 40x microscope objective (NA 0.75), and holograms are recorded with a Mach-Zehnder interferometer illuminated with a laser diode (780 nm) in an off-axis configuration. QPI images are retrieved through Fourier filtering^44^. Cells in the field of view are excited with a continuous-wave 532 nm laser (170 mW/*μ*m^2^), and the back-scattered light is separated from the excitation with a dichroic mirror, and sent into a spectrometer to measure the vibrational spectrum (535–3075 cm^−1^) with a cooled scientific CMOS detector (exposure time 3 s).

Auto-fluorescence images are acquired in a standard epi-fluorescence configuration, where the light from a mercury lamp is filtered with a DAPI filter set and sent onto the sample with a set of flipping mirrors. The fluorescence is then recorded with a scientific CMOS detector (exposure 100 ms).

### Data analysis

For each image, cells are extracted from the field of view through segmentation with the *CellProfiler* program. QPI and AF images are then registered, and morphological features are extracted from all cells with the modules providing parameters for size, shape, intensity, radial distribution and texture^29^, yielding a morphological vector of 301 parameters (a list of the complete set of features can be found in Table S3). Raman spectra are first baseline corrected with cubic spline interpolation and the silent region (1800–2700 cm^−1^) is removed. Spectra from different days are calibrated by interpolating them on a common grid based on a spectrum of pure ethanol measured each day.

Statistical analysis is performed with the *R* program^45^. Principal component analysis is performed on the data pre-scaled by variance with the *prcomp* function, and F-test values are obtained with analysis of variance (*aov*). Penalised logistic regression is performed with the *glmnet* package, where the penalisation factor that reduces the amount of variables used for classification is adjusted manually based on the results obtained with 10-fold cross validation.

## Supplementary information

Supplementary Document

## Data Availability

The raw data and datasets acquired and generated during the current study are available from the corresponding author on reasonable request.
