# Supplementary material for: Immune cell type, cell activation, and single cell heterogeneity revealed by label-free optical methods
Source: Sci Rep. 2019 Nov 19;9:17054. doi: 10.1038/s41598-019-53428-3 (PMC6864054; doi:10.1038/s41598-019-53428-3)
Supplement: Supplementary file 1 — Supplementary Document [file 41598_2019_53428_MOESM1_ESM.pdf]

Immune cell type, cell activation, and single cell heterogeneity revealed by label-free optical methods

Nicolas Pavillon[1,*] **and Nicholas I. Smith**[1,+]

[1]Biophotonics Laboratory, Immunology Frontier Research Center (IFReC), Osaka University, Yamadaoka 3-1, Suita, 565-0871, Suita, Osaka, Japan
[*]n-pavillon@ifrec.osaka-u.ac.jp
[+]nsmith@ap.eng.osaka-u.ac.jp

Supplementary information

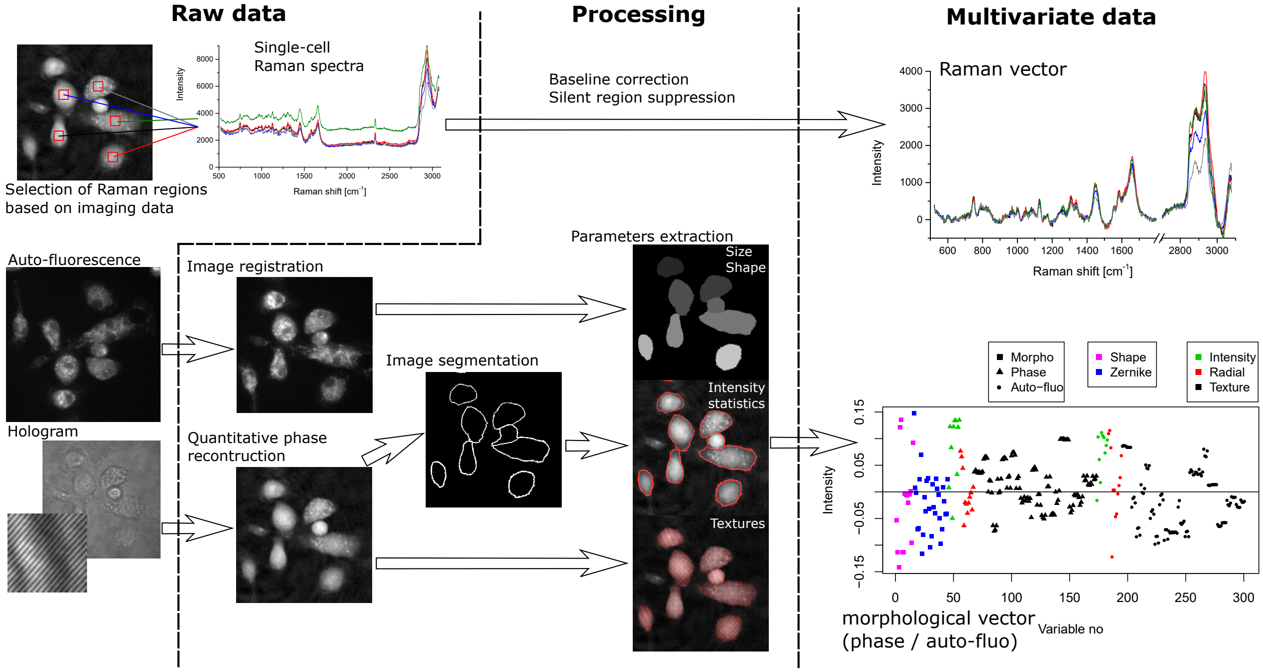

Figure S1. Summary of the data processing step, from the Raw data (Raman spectra and images) to the single-cell multivariate vectors for both Raman and morphological data.

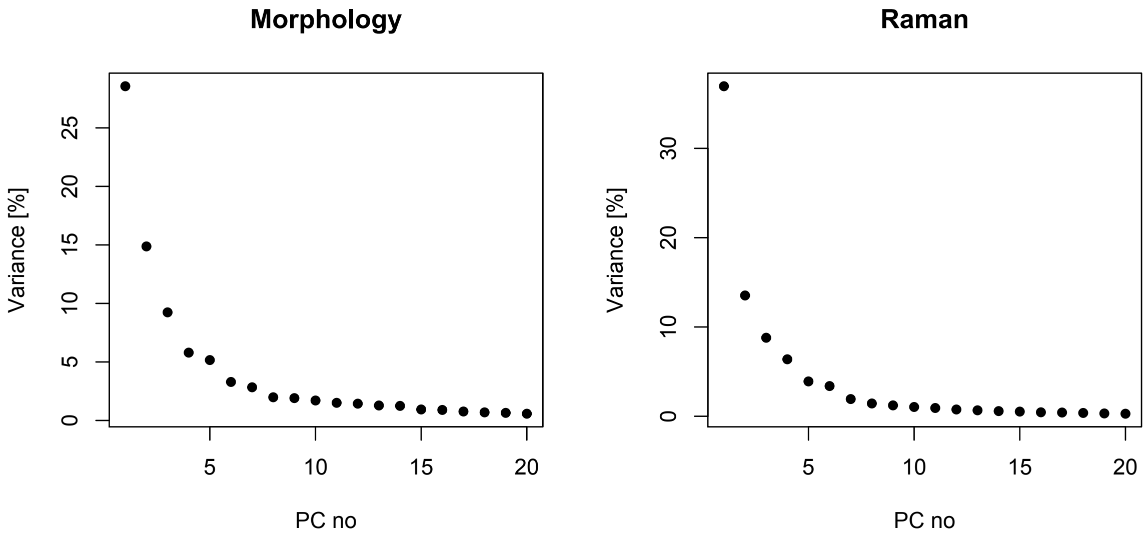

Figure S2. Variance contribution of each PC for morphology and Raman variables, respectively.

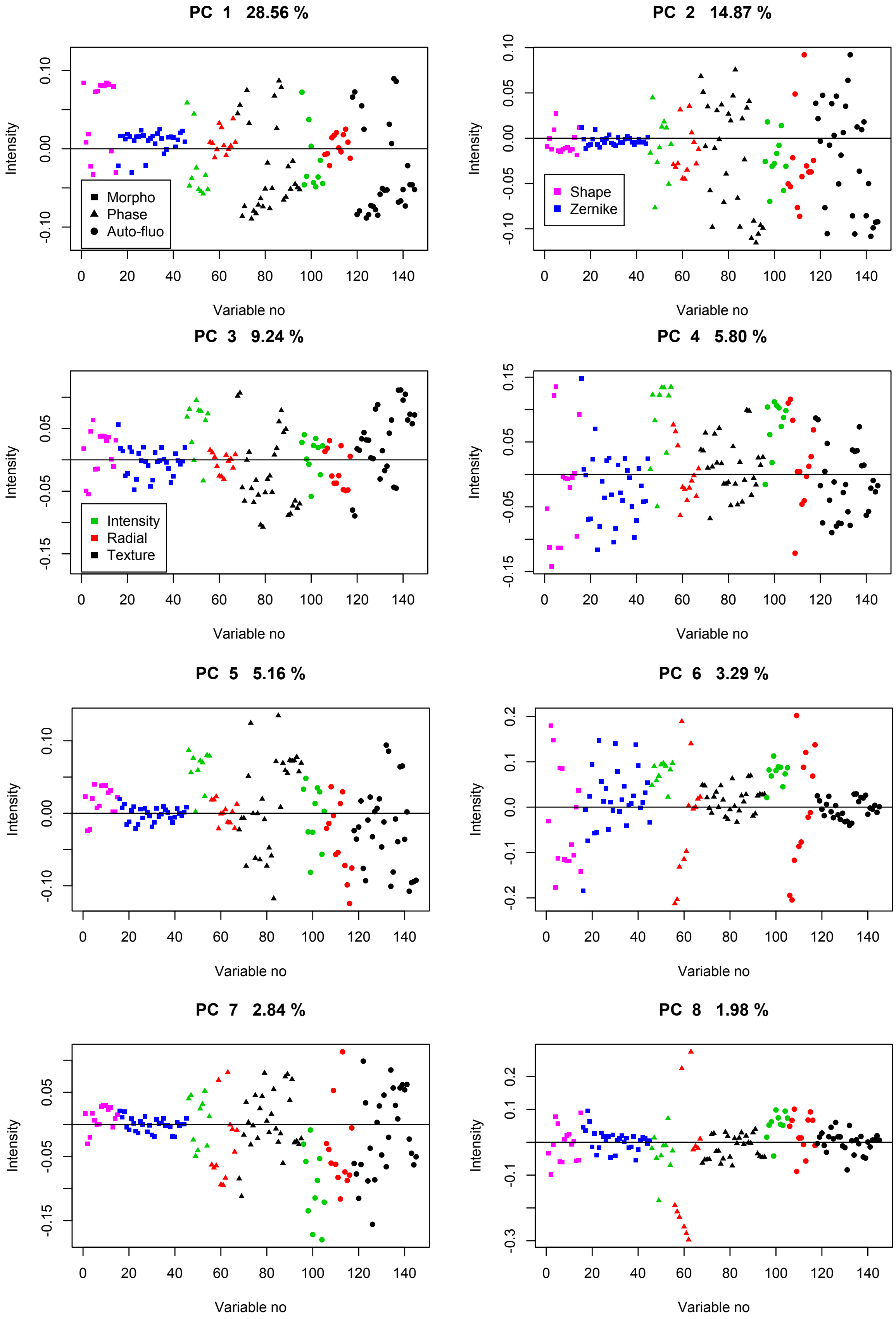

Figure S3. Morphology loading vectors 1–8 from PCA. Variance contribution for each PC is indicated.

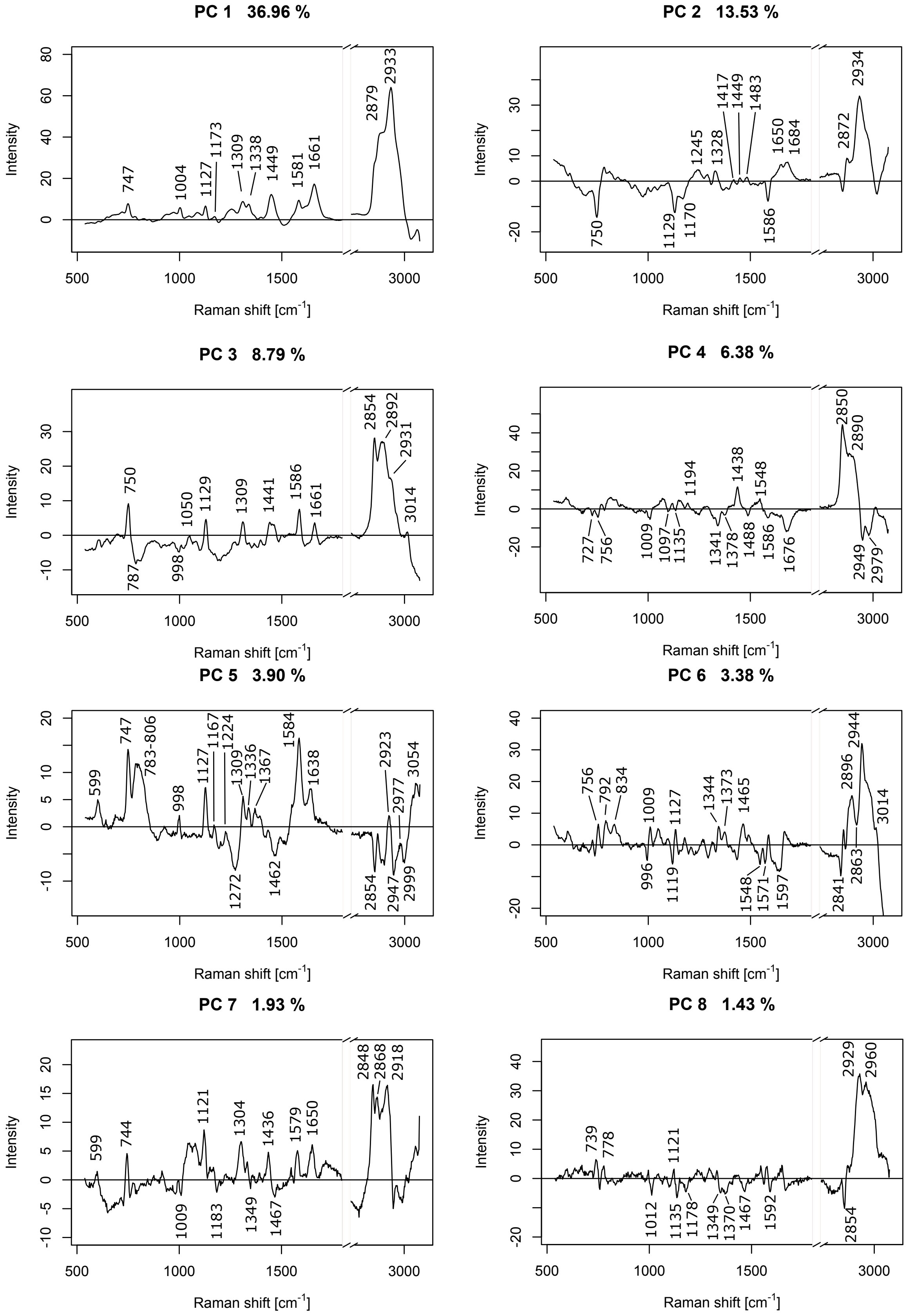

Figure S4. Raman loading vectors 1–8 from PCA with the values of the main peaks. Variance contribution for each PC is indicated.

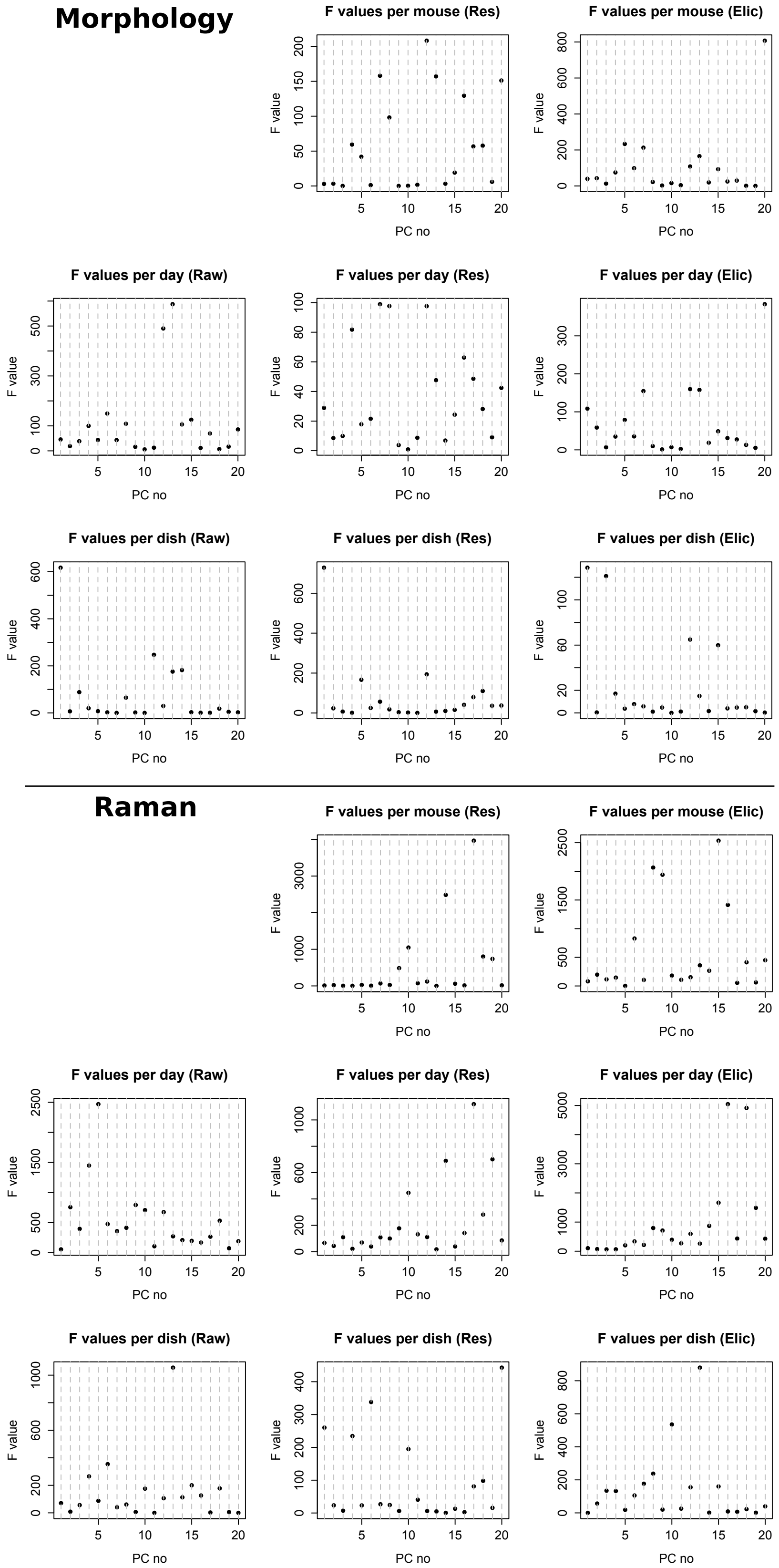

Figure S5. F-test values computed for days, mice and dishes, separately for each cell type, for morphology and Raman, respectively.

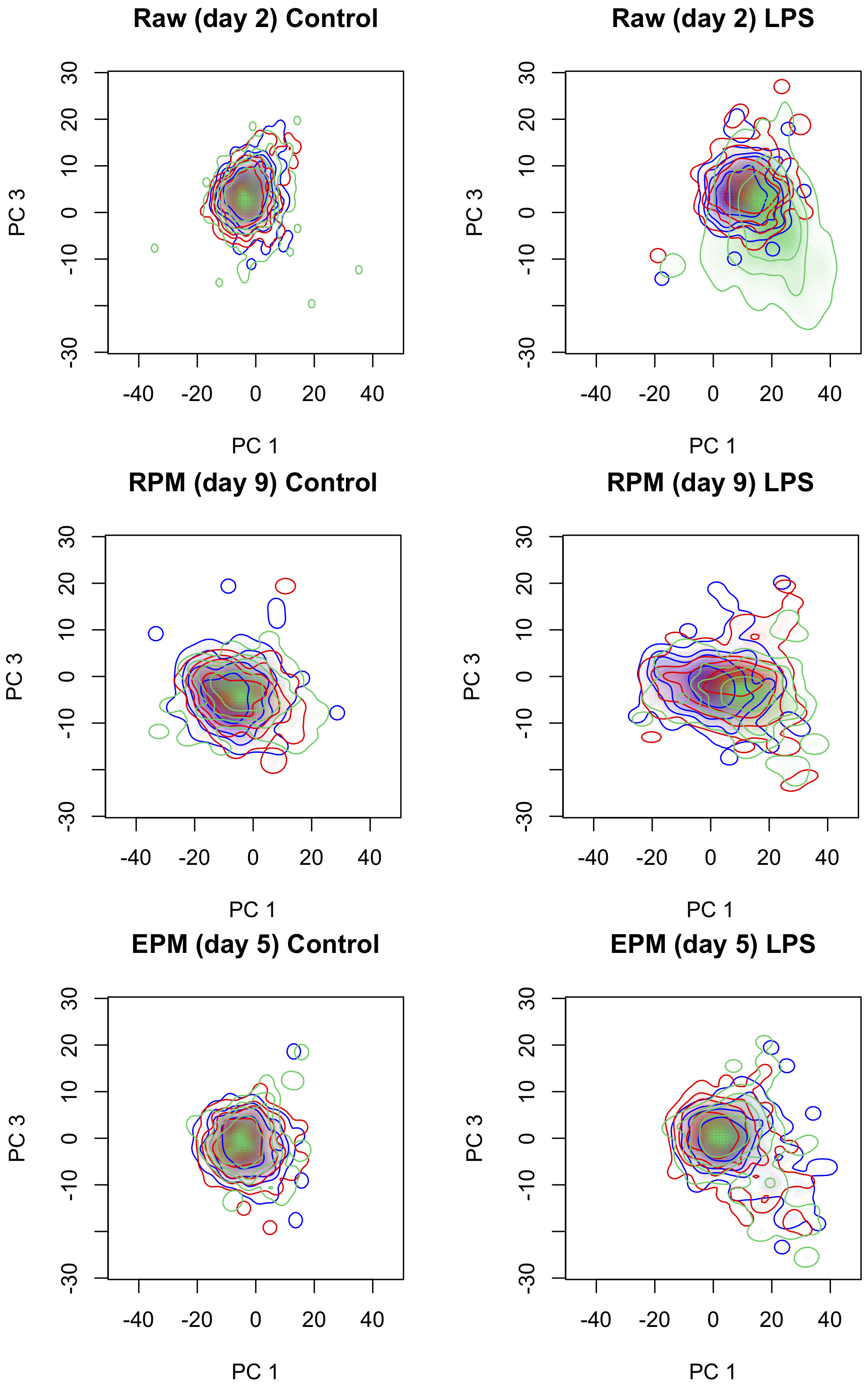

Figure S6. Influence of dishes on morphological features shown in the density maps of the score plots for PC1,3 separated per cell types and conditions. Each colour represents a separate dish.

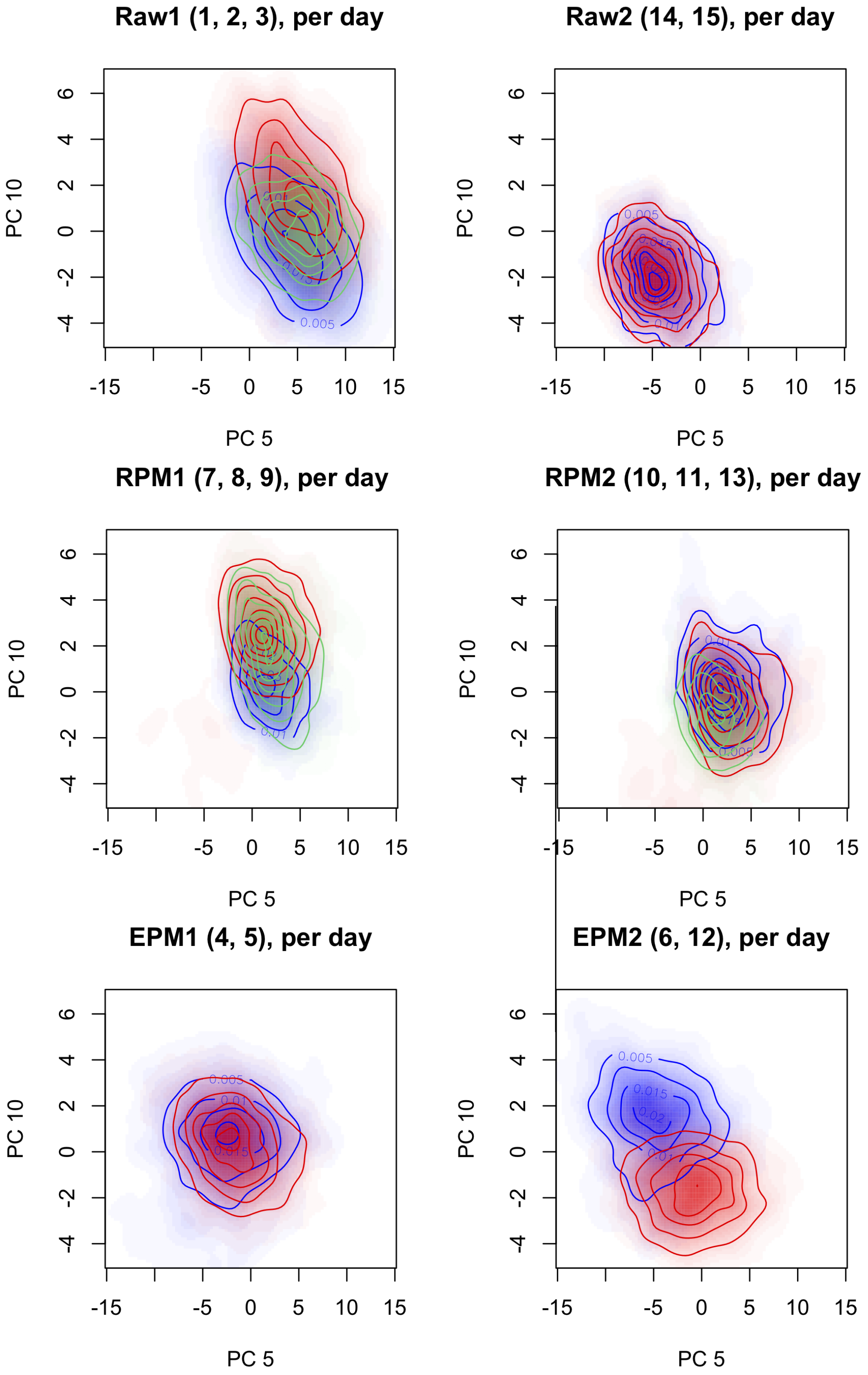

Figure S7. Influence of days on Raman features shown in the density maps of the score plots for PC5,10 separated per cell types. Each colour represents a separate day.

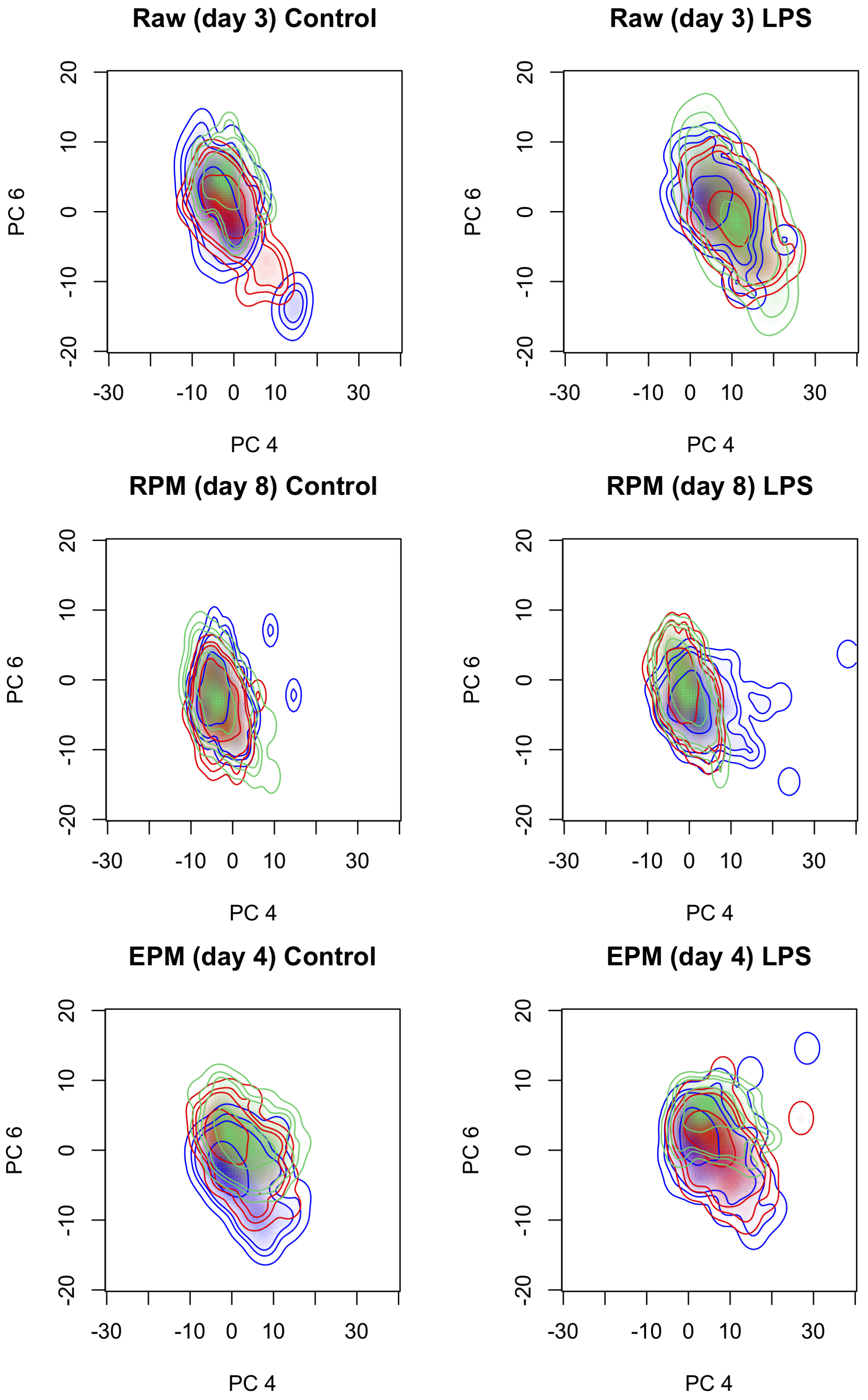

Figure S8. Influence of dishes on Raman features shown in the density maps of the score plots for PC4,6 separated per cell types and conditions. Each colour represents a separate dish.

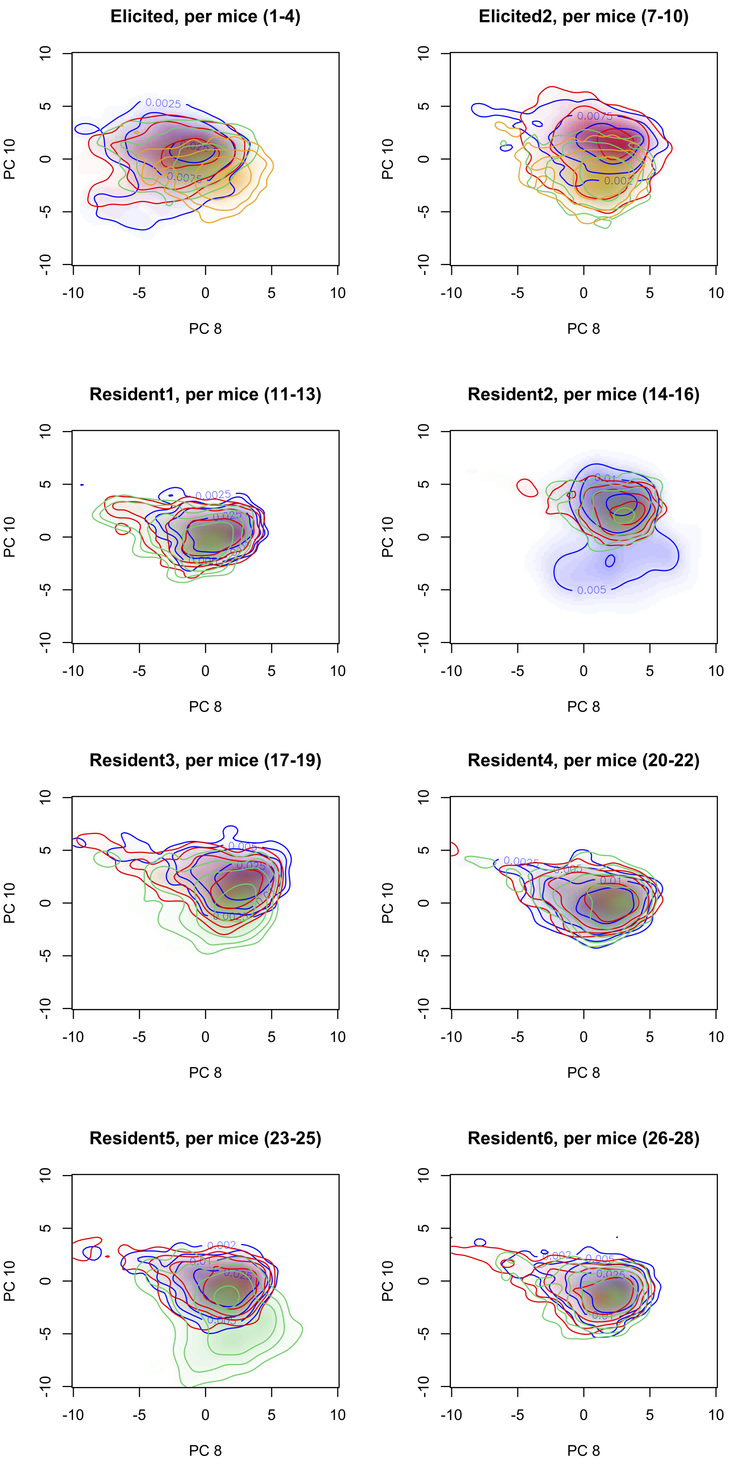

Figure S9. Density maps of the score plots from Raman parameters where the influence on mice can be identified, plotted by cell type and day, with all experimental days for resident and elicited cells (see Fig. 6)

N=7'507		Raw		Resident		Elicited	
		Control	LPS	Control	LPS	Control	LPS
Raw	Control	98.90	1.44	0.00	0.00	0.00	0.00
	LPS	0.313	99.18	0.00	0.059	0.00	0.069
Resident	Control	0.157	0.206	93.79	5.30	0.724	0.206
	LPS	0.00	0.206	4.45	94.41	0.263	0.960
Elicited	Control	0.00	0.412	1.289	0.471	91.71	6.45
	LPS	0.00	0.617	0.059	1.590	5.79	91.84

Table S1. Confusion matrices for classification predictions with paired Raman/morphology data.

Experiment no	Date	Cell type	Mice no
1	2017.12.05	Raw	-
2	2017.12.07	Raw	-
3	2017.12.12	Raw	-
4	2018.04.17	Elicited	1, 2
5	2018.04.20	Elicited	3, 4
6	2018.04.27	Elicited	7, 8
7	2018.05.02	Resident	11, 12, 13
8	2018.07.09	Resident	14, 15, 16
9	2018.07.10	Resident	17, 18, 19
10	2018.07.16	Resident	20, 21, 22
11	2018.07.17	Resident	23, 24, 25
12	2018.07.27	Elicited	9, 10
13	2018.08.01	Resident	26, 27, 28
14	2018.08.15	Raw	-
15	2018.08.21	Raw	-

Table S2. Experiment log, showing the cell type and the amount of animals used each day.

No	Type	No	Type
	Size, shape		Texture
1	Area	69–72	AngularSecondMoment / 10 px
2	Compactness	73–76	——— / 3 px / 0,135,45,90°
3	Eccentricity	77–80	Contrast / 10 px
4	EulerNumber	81–84	——— / 3 px / 0,135,45,90°
5	Extent	85–88	Correlation / 10 px
6	FormFactor	89–92	——— / 3 px / 0,135,45,90°
7	MajorAxisLength	93–96	DifferenceEntropy / 10 px
8	MaxFeretDiameter	97–100	——— / 3 px / 0,135,45,90°
9	MaximumRadius	101–104	DifferenceVariance / 10 px
10	MeanRadius	105–108	——— / 3 px / 0,135,45,90°
11	MedianRadius	109–112	Entropy_10_0
12	MinFeretDiameter	113–116	——— / 3 px / 0,135,45,90°
13	MinorAxisLength	117	Gabor / 10 px
14	Orientation	118	——— / 3 px
15	Perimeter	119–122	InfoMeas1 / 10 px
16	Solidity	123–126	——— / 3 px / 0,135,45,90°
17–46	Zernike coefficients	127–130	InfoMeas2 / 10 px
	Intensity	131–134	——— / 3 px / 0,135,45,90°
47	IntegratedIntensity	135–138	InverseDifferenceMoment 10 px
48	LowerQuartileIntensity	139–142	——— / 3 px / 0,135,45,90°
49	MADIntensity	143–146	SumAverage / 10 px
50	MassDisplacement	147–150	——— / 3 px / 0,135,45,90°
51	MaxIntensity	151–154	SumEntropy / 10 px
52	MeanIntensity	155–158	——— / 3 px / 0,135,45,90°
53	MedianIntensity	159–162	SumVariance / 10 px
54	MinIntensity	163–166	——— / 3 px / 0,135,45,90°
55	StdIntensity	167–170	Variance / 10 px
56	UpperQuartileIntensity	171–174	——— / 3 px / 0,135,45,90°
	Radial distribution		
57–60	FracAtD 1–4	175–	*Repeat Intensity, Radial, Texture*
61–64	MeanFrac 1–4	301	*for AF images*
65–68	RadialCV 1–4		

Table S3. List of morphological parameters extracted from both QPI and AF images by built-in modules from the *CellProfiler* program.
